# Evaluation of 5-methylcytosine and 5-hydroxymethylcytosine levels in juvenile idiopathic arthritis and its types

**DOI:** 10.1590/1806-9282.20250181

**Published:** 2025-09-19

**Authors:** Seyda Dogantan, Burcu Bozkaya Yücel, Sevde Hasanoğlu Sayin, Sema Nur Taskin, Yasemin Oyaci, Sacide Pehlivan

**Affiliations:** 1 Başakşehir Çam and Sakura City Hospital, Department of Pediatric Rheumatology – Istanbul, Turkey.; 2 Samsun Education and Research Hospital, Department of Pediatrics – Samsun, Turkey.; 3 Bezmialem Vakıf University, Experimental Application and Research Center – Istanbul, Turkey.; 4 Eskisehir State Hospital, Department of Pediatric Rheumatology – Eskişehir, Turkey.; 5 Istanbul University, Istanbul Medical Faculty, Department of Medical Biology – Istanbul, Turkey.

**Keywords:** Juvenile idiopathic arthritis, Epigenetic, 5-mC, 5-methylcytosine, DNA methylation, 5-hydroxymethyl cytosine, ELISA, DNA

## Abstract

**OBJECTIVE:**

Juvenile idiopathic arthritis is the most common chronic rheumatic disease of autoimmune origin in childhood. While altered DNA methylation has been associated with adult autoimmune rheumatic diseases, studies on juvenile idiopathic arthritis remain limited. This study evaluated levels of 5-methylcytosine and 5-hydroxymethylcytosine, important epigenetic biomarkers, in juvenile idiopathic arthritis and its subtypes.

**METHODS:**

This single-center cross-sectional study included patients diagnosed with juvenile idiopathic arthritis aged 1 month to 18 years. Children with systemic or autoimmune diseases and comorbidities were excluded. The study comprised 58 juvenile idiopathic arthritis patients and 35 age-gender-matched healthy controls presenting with non-rheumatological complaints. Demographic and clinical characteristics, including juvenile idiopathic arthritis subtypes, were recorded. The 5-methylcytosine and 5-hydroxymethylcytosine levels were assessed quantitatively using an enzyme-linked immunosorbent assay kit. The relationships between 5-methylcytosine and 5-hydroxymethylcytosine and juvenile idiopathic arthritis and its subtypes were evaluated statistically.

**RESULTS:**

The 5-hydroxymethylcytosine level was significantly higher, whereas the 5-methylcytosine and 5-methylcytosine/5-hydroxymethylcytosine ratios were significantly lower in the patient group than in the control group. No significant differences between different juvenile idiopathic arthritis subtypes and disease activity status were found in 5-hydroxymethylcytosine and 5-methylcytosine levels or 5-methylcytosine/5-hydroxymethylcytosine ratios (p>0.05). The 5-methylcytosine/5-hydroxymethylcytosine ratio, with an optimal cut-off value of ≤28.05, demonstrated a sensitivity of 70.69% and a specificity of 77.14% in distinguishing juvenile idiopathic arthritis patients from controls (p<0.001).

**CONCLUSION:**

5-Hydroxymethylcytosine and 5-methylcytosine can be potential biomarkers for juvenile idiopathic arthritis and its subtypes, indicating their roles in juvenile idiopathic arthritis development regarding molecular mechanisms and novel therapeutic approaches.

## INTRODUCTION

Juvenile idiopathic arthritis (JIA) is a complex childhood autoimmune rheumatic disease that affects children aged 6 months to 16 years^1^. Literature data suggest that several immune system components, such as inflammatory cells, including neutrophils, plasma cells, dendritic cells, and activated T-cells, are involved in the pathogenesis of JIA. Although the exact cause of JIA is unknown, family studies indicate that it is a complex disease in which environmental and genetic variables play significant roles^2^.

Global DNA methylation via the insertion of a methyl at position 5 of cytosine in cytosine-phosphate-guanine (CpG) dinucleotides, resulting in the formation of 5-methylcytosine (5-mC), which affects gene expression, chromatin remodeling, and genome integrity, might have a role in understanding physiological mechanisms and disease conditions^3,4^. Transformation of 5-mC into 5-hydroxymethylcytosine (5-hmC) may influence various intracellular functions^5^. Imbalances in 5-mC and 5-hmC markers cause transcriptional dysregulation of enhancers and promoters, altering the cell's fate^6^. Aberrant DNA methylation is also implicated in several autoimmune diseases, including rheumatoid arthritis^7^.

This study aimed to investigate the 5-mC and 5-hmC levels in JIA patients and healthy controls and to analyze the impact of JIA subtypes and disease activity on methylation biomarkers.

## METHODS

### Study design

The study was designed as a single-center cross-sectional study. The protocol was approved by the Non-Interventional Clinical Research Ethics Committee of Samsun Training and Research Hospital (GOKAEK 2024/23/16). The study was conducted according to the ethical considerations outlined in the Declaration of Helsinki. Informed consent was obtained from the parents or legal guardians of the children included in the study. Children's consent was also obtained to ensure that they understood the purpose and procedures of the study.

### Population and sample

The study population comprised patients between 1 month and 18 years diagnosed with JIA according to the International League of Associations for Rheumatology criteria^8^. Patient data were retrospectively collected from two centers: the Department of Pediatric Rheumatology at Başakşehir Çam and Sakura City Hospital in Istanbul and Samsun University Training and Research Hospital in Samsun, Turkey. The study was conducted under ethical approval obtained from Samsun Training and Research Hospital. Children with any systemic or autoimmune diseases and comorbidities were excluded from the study. In the end, the patient group consisted of 58 patients with JIA, and the control group consisted of 35 age- and gender-matched healthy children who presented to the outpatient clinics of the same hospital with complaints unrelated to rheumatological conditions.

### Data collection

Children's demographic and clinical characteristics, including disease duration, disease activity status, JIA subtype classification, and biological treatments and inflammatory marker levels (C-reactive protein [CRP] and erythrocyte sedimentation rate [ESR]), were recorded.

### DNA isolation

Three milliliters of blood were collected from all participants, regardless of disease status, into tubes containing ethylenediaminetetraacetic acid upon their admission to the pediatric rheumatology outpatient clinics. Blood samples from healthy controls were obtained during the same study period.

Genomic DNA was extracted from blood samples taken from the participants using ELK Biotechnology's Blood/Cell/Tissue Genomic DNA Extraction Kit (Cat no: EP007), following the manufacturer's instructions. The extracted DNA samples were stored at −20°C.

### 5-Methylcytosine measurements

For 5-mC global DNA methylation, the Abcam Global DNA Methylation Assay Kit (5-Methyl Cytosine, Colorimetric, cat no: ab233486) was used. The rate of methylated DNA (5-mC) in the total DNA was calculated using the following formula: 5-mC%=sample's optical density (OD)-negative control's OD×100%: slope ×S.

### 5-Hydroxymethylcytosine measurements

For 5-hmC global DNA hydroxymethylation, the Abcam Global DNA Hydroxymethylation Assay Kit (5-hydroxymethyl Cytosine, Colorimetric, cat no: ab233487) was used. The percentage of hydroxymethylated DNA (5-hmC) in the total DNA was calculated using the following formula: 5-hmC%=sample's OD-negative control's OD×100%: slope ×S.

### Statistical analysis

Statistical analyses were conducted using the JAMOVI software (Version 2.3.28, The Jamovi Project, 2023, https://www.jamovi.org) and JASP (Version 0.19.0, Jeffreys’ Amazing Statistics Program, 2024, https://jasp-stats.org). Given the sample sizes in our JIA (n=58) and control (n=35) groups, we employed the Kolmogorov-Smirnov test for the JIA group and the Shapiro-Wilk test for the control group to assess normality distribution, complemented by visual examination of histograms and Q-Q plots.

The results of the statistical analyses were expressed using descriptive statistics, that is, median values for quantitative variables and numbers for categorical variables.

The differences between the 5-mC global methylation and 5-hmC global hydroxymethylation results of the patient and control groups were assessed using the independent samples median test and Fisher's exact test, where necessary. The relationships between global methylation, hydroxymethylation levels, and clinical parameters were evaluated using the Spearman rank correlation (ρ) test. Probability (p) statistics of <0.05 were deemed to indicate statistical significance.

To evaluate the 5-mC/5-hmC ratio's diagnostic potential in distinguishing JIA patients from controls, receiver operating characteristic (ROC) curve analysis was performed using DeLong's method. The calculation of area under the curve (AUC), sensitivity, specificity, and optimal cut-off value was determined by the Youden index (J=sensitivity+specificity—1), where a larger value indicates better diagnostic performance.

Height, weight, and body mass index (BMI) Z-scores were calculated using the 2000 Centers for Disease Control and Prevention (CDC) growth charts as reference values.

## RESULTS

The distribution of the demographic and clinical characteristics by the patient and control groups is shown in Table 1. Eight patients (13.8%) had active disease at the time of blood sampling. Accordingly, the most common (34.5%) JIA subtype was oligoarticular JIA, and uveitis was present in four (6.9%) patients. In terms of biological treatments JIA patients received, adalimumab was used most frequently (58.6%), followed by etanercept (20.7%) and tocilizumab (10.3%). No statistically significant differences were observed between patient and control groups regarding height Z-scores (p=0.210), weight Z-scores (p=0.183), and BMI Z-scores (p=0.157) based on CDC reference values.

**Table 1:** Demographic and clinical characteristics of groups.

		Patients group (n=58)	Control group (n=35)	p*
Age (years)§		14.0 [4.0–18.0]	13.0 [10.0–18.0]	0.702*
Gender‡				
	Female	32 (55.2)	20 (57.1)	0.999**
	Male	26 (44.8)	15 (42.9)	
Height (cm)§		153.5 [102.0–180.0]	159.0 [132.0–176.0]	0.243*
Weight (kg)§		46.5 [16.0–135.0]	50.0 [25.0–74.0]	0.140*
Body mass index (kg/m)§		19.0 [13.0–46.0]	21.2 [14.0–28.0]	0.168*
Height Z-score		0.1 [-3.7–0.9]	-0.1 [-3.2–3.1]	0.210
Weight Z-score		0.5 [-5.8–1.5]	0.0 [-2.8–3.6]	0.183
Body mass index Z-score		0.7 [-3.9–1.7]	0.2 [-3.2–2.9]	0.157
Disease activity‡				
	Inactive	50 (86.2)	–	–
	Active	8 (13.8)	–	–
Disease duration (years)§		2.0 (1.0–15.0)	–	–
Disease type‡			–	–
	Oligoarticular JIA	20 (34.5)	–	–
	Enthesitis-related arthritis	19 (32.8)	–	–
	Polyarticular JIA	10 (17.2)	–	–
	Systemic JIA	5 (8.6)	–	–
	Psoriatic arthritis	4 (6.9)	–	–
Uveitis, present‡		4 (6.9)	–	–
Drugs used‡			–	–
	Adalimumab	34 (58.6)	–	–
	Etanercept	12 (20.7)	–	–
	Tocilizumab	6 (10.3)	–	–
	Canakinumab	4 (6.9)	–	–
	Anakinra	1 (1.7)	–	–
	Infliximab	1 (1.7)	–	–
Biomarker and inflammatory parameters				
	5-hmC (%)§	0.03 [0.01–0.23]	0.02 [0.01–0.06]	**<0.001**
	5-mC (%)§	0.67 [0.26–1.36]	0.84 [0.48–1.08]	**0.038**
	5-mC/5-hmC§	22.9 [4.0–54.0]	38.2 [14.8–89.7]	<0.001
	CRP (mg/L)§	3.0 [1.0–126.0]	1.2 [0.1–40.1]	**<0.001**
	ESR (mm/hr)§	7.0 [2.0–77.0]	7.0 [2.0–65.0]	0.830

* Mann-Whitney U test.

** Pearson chi-square test.

‡ n (%);

§ Median [min–max].

JIA: juvenile idiopathic arthritis, CRP: C-reactive protein, ESR: erythrocyte sedimentation rate; 5-mC: 5-methylcytosine; 5-hmC: 5-hydroxymethylcytosine. **Kruskal-Wallis test. Bold p-values indicate statistical significance (p≤0.05).

**Figure 1:**
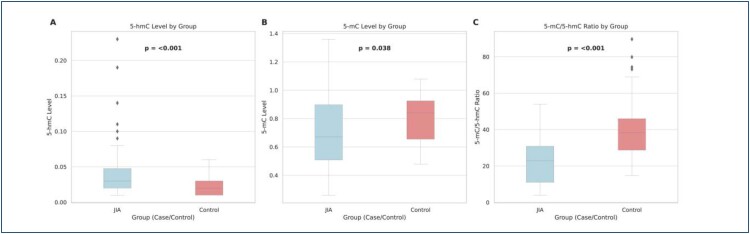
Comparison of DNA methylation and hydroxymethylation levels between juvenile idiopathic arthritis patients and healthy controls. Box plots showing (A) 5-hydroxymethylcytosine levels, (B) 5-methylcytosine levels, and (C) 5-methylcytosine/5-hydroxymethylcytosine ratios in juvenile idiopathic arthritis patients (n=58) versus healthy controls (n=35). Juvenile idiopathic arthritis patients demonstrated significantly higher 5-hydroxymethylcytosine levels (p<0.001), significantly lower 5-methylcytosine levels (p=0.038), and significantly lower 5-methylcytosine/5-hydroxymethylcytosine ratios (p<0.001) compared to controls. Box plots display median values with interquartile ranges; individual data points represent outliers. Statistical significance was determined using independent samples median test.

The distribution of biomarker and inflammatory parameters by the patient and control groups is shown in Table 1. The 5-hmC level was significantly higher, whereas the 5-mC level and 5-mC/5-hmC ratio were significantly lower in the patient group compared to controls (p<0.001, p=0.038, and p<0.001, respectively) (Figure 1). Regarding inflammatory markers, CRP level was significantly higher in patients (p<0.001), while ESR showed no significant difference between groups (p>0.05).

There was no significant difference in 5-hmC and 5-mC levels and, thus, in the 5-mC/5-hmC ratio between patients with different JIA subtypes (p>0.05) (Table 2).

**Table 2:** Comparison of 5-methylcytosine, 5-hydroxymethylcytosine levels, and 5-methylcytosine/5-hydroxymethylcytosine ratio according to disease activity in juvenile idiopathic arthritis patients.

Parameter	Oligoarticular JIA (n=20)	Enthesitis-related arthritis (n=19)	Subtypes	Systemic JIA (n=5)	Psoriatic arthritis (n=4)	p**	Disease activity		p*
			Polyarticular JIA (n=10)				Inactive JIA (n=50)	Active JIA (n=8)	
5-hmC (%)§	0.0 [0.0–0.1]	0.0 [0.0–0.2]	0.1 [0.0–0.2]	0.0 [0.0–0.0]	0.0 [0.0–0.1]	0.177	0.0 [0.0–0.2]	0.0 [0.0–0.1]	0.228
5-mC (%)§	0.6 [0.3–1.2]	0.8 [0.4–1.1]	0.7 [0.5–1.4]	0.5 [0.3–1.4]	0.8 [0.6–1.1]	0.531	0.6 [0.3–1.4]	0.7 [0.5–1.4]	0.195
5-mC/5-hmC ratio§	20.0 [5.3–38.6]	23.1 [4.4–54.0]	14.6 [4.0–44.5]	43.3 [11.9–50.1]	27.0 [10.5–45.2]	0.246	23.4 [4.0–50.1]	15.8 [6.6–54.0]	0.752

* Mann-Whitney U test.

** Kruskal-Wallis test.

§ Median [min–max].

JIA: juvenile idiopathic arthritis; 5-mC: 5-methylcytosine; 5-hmC: 5-hydroxymethylcytosine.

As shown in Table 2, no significant differences were observed in 5-hmC levels, 5-mC levels, or 5-mC/5-hmC ratios between patients with active and inactive JIA (p>0.05 for all comparisons).

The ROC curve analysis revealed that the 5-mC/5-hmC ratio demonstrated good diagnostic performance in distinguishing JIA patients from controls, with an AUC of 0.760 (95%CI 0.660–0.842, p<0.001), sensitivity of 70.69%, and specificity of 77.14% at the optimal cut-off value of ≤28.05.

## DISCUSSION

JIA involves synovial tissue containing various inflammatory cells, including neutrophils, plasma cells, dendritic cells, and most activated T cells^2^. It has been suggested that the highly activated Th1-type phenotype of T cells in chronically inflamed joints of children with JIA may reflect specific recruitment events that contribute to the polarization of these cells.^9^. These T-cells produce tumor necrosis factor beta, interleukin 2, and interferon-γ. Th1-dominant responses have been linked to several other autoimmune conditions, including psoriasis, inflammatory bowel disease, rheumatoid arthritis (RA), and type 1 diabetes mellitus^2^. Although the etiology of autoimmune disorders involves a complex genetic predisposition^10^, environmental variables also play significant roles in the development of autoimmune diseases, as demonstrated in genetically identical monozygotic twins^11^.

Examples of epigenetic mechanisms and elements affected by hereditary and environmental variables include DNA methylation, post-transcriptional modifications of histones, and microribonucleic acids^12^. These mechanisms and elements control gene transcription rates, modify chromatin architecture, and govern accessibility to transcriptional regulatory factors. The most extensively researched and thoroughly described epigenetic alteration mechanism is DNA methylation. The DNA methyltransferase (DNMT)-mediated enzymatic transfer of a methyl group from S-adenosyl-L-methionine to the CpG dinucleotide results in in the formation of 5-mC. The CpG dinucleotides, which are not typical and severely methylated in the bulk genome, are typically unmethylated in CpG island clusters, which are collections of CpG dinucleotides. Methylation of CpG islands in gene promoters is linked to the long-term suppression of gene expression. On the other hand, tissue-specific and variable methylation preferentially occurs outside of CpG islands. In a study investigating the transcript levels of types DNMT1, DNMT3A, and DNMT3B in peripheral blood mononuclear cells to assess the importance of epigenetic changes in the pathogenesis of JIA, Ghavidel et al. found that DNMT1 and DNMT3A expressions were significantly lower in JIA patients than in healthy control subjects (7- and 5.5-fold, respectively), and this difference was even more significant in the case of young male JIA patients^13^.

Several inflammatory and autoimmune disorders have been linked to global DNA hypomethylation, which involves abnormal gene and ribosomal RNA expression that likely plays a role in the disease's pathophysiology^14^. Autoimmune diseases have not been linked to other DNA hypomethylation-related alterations, such as genomic instability, mutations, or the use of cryptic promoters^15^. It has been demonstrated that systemic lupus erythematosus (SLE) cluster of differentiation 4 (CD4+) T cells have global DNA hypomethylation, contributing to the over-reactivity of T cells in SLE patients^16,17^. SLE CD4+ T cells have lower levels of DNMT1, an enzyme that maintains DNA methylation, indicating that passive DNA demethylation contributes to the pathophysiology of SLE^18^. Fibroblast-like synoviocytes (FLS) in RA have been shown to exhibit DNA hypomethylation. Overexpression of genes essential to the disease process has been linked to hypomethylation at particular CpG sites in FLS. The active phenotype typical of FLS develops following 5-azacytidine-induced demethylation, highlighting the effects of DNA hypomethylation in FLS. However, there are still many unresolved aspects of DNA methylation in RA. DNA methylation in the gene encoding the pro-inflammatory cytokine interleukin-32 is decreased in CD4+ T cells of JIA patients^7^. All these findings suggest that unmethylated cytokine production increases and upregulates immune system hyperactivity, potentially leading to autoimmune diseases. Hence, decreased DNA methylation is likely linked to the pathogenesis of JIA.

Based on these literature data, we investigated the peripheral blood 5-mC and 5-hmC levels in JIA patients and the relationships between DNA methylation and hydroxymethylation and JIA subtypes. Consequently, we found that the 5-hmC level was significantly higher. In contrast, the 5-mC level and, thus, the 5-mC/5-hmC ratio were significantly lower in the patients with JIA than in healthy control subjects, suggesting that epigenetic changes play a role in JIA pathogenesis.

Our ROC curve analysis highlighted the potential diagnostic utility of the 5-mC/5-hmC ratio as a novel biomarker for JIA, demonstrating good discriminatory power between patients and healthy controls. We observed balanced sensitivity and specificity, suggesting its potential value as a supplementary diagnostic tool. These findings are particularly noteworthy given the current challenges in early JIA diagnosis and the need for objective biomarkers. The relatively high specificity suggests that the 5-mC/5-hmC ratio could be especially valuable in confirming disease when used with traditional clinical and laboratory parameters. However, the moderate sensitivity indicates that this marker alone may not be sufficient for screening. Future longitudinal studies should investigate whether this epigenetic ratio could also serve as a predictive marker for disease progression or treatment response, potentially contributing to more personalized therapeutic approaches in JIA management.

We also found that these epigenetic modifications were associated with elevated CRP levels. However, we did not find any significant relationship between these epigenetic changes and JIA subtypes, which may be attributed to the insufficient patients in the JIA subgroups.

### The limitations of the study

The primary limitation of our study was that the cross-sectional design may not have allowed for the establishment of causal relationships between epigenetic changes and disease progression. Secondly, insufficient patients in the JIA subgroups may have limited our ability to detect JIA subtype-specific methylation differences. Our study does not exclude the need to investigate gene-specific methylation alterations. Lastly, the lack of pre-treatment methylation data and the differences between the treatment regimens received by patients prevented us from drawing any conclusions about the impact of current medical treatments on methylation patterns. Given these limitations of our study, caution is advised when interpreting its results.

## CONCLUSION

Although the etiology of JIA is not fully known, the interaction between genes and environment is likely to be important, as in other autoimmune diseases. In this context, a critical finding of our study is that the 5-hmC levels were elevated in patients with JIA, highlighting a key aspect of epigenetic mechanisms influencing inflammatory processes in JIA. In conclusion, 5-hmC and 5-mC can be potential biomarkers of JIA and its subtypes. The study's findings regarding the roles of 5-hmC and 5-mC in the development of JIA pave the way for further studies to elucidate the pathogenesis of JIA and develop new treatment options.

## Data Availability

The datasets generated and/or analyzed during the current study are available from the corresponding author upon reasonable request.
